# In Situ Distribution of HIV-Binding CCR5 and C-Type Lectin Receptors in the Human Endocervical Mucosa

**DOI:** 10.1371/journal.pone.0025551

**Published:** 2011-09-30

**Authors:** Taha Hirbod, Tove Kaldensjö, Kristina Broliden

**Affiliations:** Department of Medicine, Division of Infectious Diseases, Center for Molecular Medicine, Karolinska Institutet, Karolinska University Hospital, Stockholm, Sweden; University of Rochester, United States of America

## Abstract

The endocervical mucosa is believed to be a primary site of HIV transmission. However, to date there is little known about the distribution of the HIV co-receptor CCR5 and the HIV-binding C-type lectin receptors, including Langerin, dendritic cell (DC)-specific intercellular adhesion molecule-grabbing non-integrin (DC-SIGN) and mannose receptor (MR) at this site. We therefore characterized the expression of these molecules in the endocervix of HIV seronegative women by computerized image analysis. Endocervical tissue biopsies were collected from women (n = 6) undergoing hysterectomy. All study individuals were diagnosed with benign and non-inflammatory diseases. CCR5+ CD4+ CD3+ T cells were found within or adjacent to the endocervical epithelium. The C-type lectin Langerin was expressed by intraepithelial CD1a+ CD4+ and CD11c+ CD4+ Langerhans cells, whereas DC-SIGN+ MR+ CD11c myeloid dendritic cells and MR+ CD68+ macrophages were localized in the submucosa of the endocervix. The previously defined immune effector cells including CD8+, CD56+, CD19+ and IgD+ cells were also found in the submucosa as well as occasional CD123+ BDCA-2+ plasmacytoid dendritic cells. Understanding the spatial distribution of potential HIV target cells and immune effector cells in relation to the endocervical canal forms a basis for deciphering the routes of HIV transmission events in humans as well as designing HIV-inhibiting compounds.

## Introduction

The predominant route of human immunodeficiency virus (HIV) infection in women is across the genital mucosa through heterosexual transmission. However, the relative susceptibility of individual mucosal sites in the female genital tract to primary HIV transmission is unknown. The influence of hormones and sexually transmitted infections as well as the exposure to microbial antigens in the seminal plasma make the endocervical mucosa a complex immunological environment. The mechanical barrier formed by the single-layered columnar epithelium of the endocervix is, even when intact, less robust for preventing the invasion of pathogens than the multi-layered squamous epithelium of the vagina and ectocervix [1]. Although the mucus in the endocervical canal provides a medium for innate immune molecules and forms a physical barrier to invading pathogens [2], it is never totally impermeable. In an ex vivo organ culture model, both cell-bound HIV and virions were temporarily trapped by the cervical mucus but could also reach the epithelial surface and penetrate to mucosal target cells [3].

In vivo studies in rhesus macaques [4], [5] and human vaginal explant studies [6] suggest that HIV target cells in the genital tract mucosa are CD4+ T cells, Langerhans cells (LCs), interstitial dendritic cells (DCs) and macrophages. In addition to conventional binding to the main receptor CD4 and the co-receptor CCR5; DCs, LCs and macrophages can bind HIV through other receptor pathways including C-type lectin receptors (CLRs) [7]. Little is known about the presence and distribution of HIV-binding CLRs such as the mannose receptor (MR) [8], [9], the DC-specific intercellular adhesion molecule-grabbing non-integrin (DC-SIGN) [10] and Langerin [11] in the human endocervix. The physiological role of these receptors when bound to HIV particles is dependent on the location, timing and concentration of the inoculum as well as on host inflammatory and hormonal factors. For example, HIV-binding to DC-SIGN impairs DC maturation, weakens T cell proliferation and mediates transmission of HIV to T cells [11] whereas immature LCs may form a barrier against HIV infection by efficiently capturing and degrading the virus through Langerin [12].

Previous studies of HIV or simian immunodeficiency virus (SIV) immune markers in the endocervical mucosa are limited and based on either flow cytometry of enzyme-digested tissues or cytobrush-obtained cervical specimens from humans [13], [14], [15], or immunohistochemical staining on tissue samples derived from rhesus macaques [16] and humans [17], [18], [19]. However, the distribution of CCR5+ CD4+ T cells or of CLR expressing antigen-presenting cell subsets has not been described in human endocervical tissue. This study was therefore designed to characterize and define these HIV-binding receptors at the single cell level in the epithelial and submucosal compartments of the human endocervix.

## Materials and Methods

### Study population and sample collection

Written informed consent was obtained from all study subjects, and ethical approval was obtained from the Regional Ethical Review Board in Stockholm. Endocervical tissue samples were obtained from six women undergoing hysterectomy for non-malignant, non-inflammatory indications (leiomyomata and adenomyosis); mean age was 47.5 years (range 39–51 years). The inclusion criteria were: HIV IgG seronegativity, no clinical symptoms of sexually transmitted infections during the prior three months and no use of hormonal therapy. The hysterectomy samples were immediately transported on ice to the pathology department, where a gynecological pathology specialist collected endocervical tissue samples. Menstrual cycle stage was determined by endometrial dating. Two study subjects were in the proliferative phase, two were in the secretory phase, and two had an inactive endometrium.

### In situ detection of cellular markers by immunostaining

The endocervical tissue samples were snap frozen in liquid nitrogen within 30 minutes of surgical removal. As previously described [20], [21], the cryopreserved tissue samples were sectioned to 8 µm, fixed in 2% formaldehyde, blocked for endogenous biotin (Biotin/Avidin Blocking Kit; Vector Laboratories, Burlingame, CA) and immunohistochemically stained. The following anti-human antibodies were used to detect the immune markers of interest: goat anti-Langerin (clone#: AF2088 diluted 1∶250), mouse anti-DC-SIGN (clone#: 120507 diluted 1∶500), goat anti-DLEC (BDCA-2 clone#: AF1376 diluted 1∶25) (R&D Systems, Minneapolis, MN), mouse anti-CD4 (clone#: SK3 diluted 1∶10), mouse-anti CD3 (clone#: SK7 diluted 1∶10), mouse anti-CD8 (clone#: SK1 diluted 1∶10), mouse anti-CD56 (clone#: MY31 diluted 1∶500), mouse anti-CD19 (clone#: HIB19 diluted 1∶500), mouse anti-MR (clone#: 19.2 diluted 1∶700), mouse anti-CD11c (clone#: B-ly6 diluted 1∶100), mouse anti-CD123 (clone#: 9F5 diluted 1∶1000), mouse anti-CD123 (clone#: 7G3 diluted 1∶10) (BD Pharmingen, Franklin Lakes, NJ), rat anti-CD4 (clone#: YNB46.1.8 diluted 1∶100), mouse anti-CD1a (clone#: NA/1/34-HLK diluted 1∶500 ) (Serotec, Düsseldorf, Germany), mouse anti-CD68 (clone#: EBM11 diluted 1∶200) (DAKO, Stockholm, Sweden), goat anti-IgD (clone#: 2030-08 diluted 1∶5000) (SouthernBiotech, Birmingham, AL) and mouse anti-CCR5 (clone#: MC-5 diluted 1∶100) (kindly provided by Professor M. Mack from the University Clinic of Regensburg, Germany). Thereafter biotinylated secondary antibodies diluted 1∶500 were added; rabbit anti-mouse, rabbit anti-goat (DAKO), goat anti-mouse (Caltag Laboratories, Invitrogen, Life Technologies Corporation, Carlsbad, CA, USA) or goat anti-rat (Vector laboratories). The staining reactions were developed brown by using diaminobenzidine tetrahydrochloride (DAB; Vector Laboratories), and nuclear counterstaining was performed with hematoxylin. Digital images were transferred from a DMR-X microscope (Leica, Wetzlar, Germany) into a computerized image analysis system, Quantimet, Q 550 IW (Leica Imaging Systems, Cambridge, UK). For one and double - fluorescent staining, endocervical tissue sections were stained as described above except that Alexa Flour 488 streptavidin (Invitrogen) or Alexa Flour 594 labeled streptavidin (Invitrogen) were used to visualize the cells of interest.

Fluorescently labeled cells were evaluated using the Qwin 550 software and a filter-free spectral confocal microscope (Leica TCS SP2 AOBS). Negative control staining consisted of irrelevant mouse, rat or goat IgG (DAKo).

### Quantitative analysis

Expression of the investigated markers was analyzed in approximately 3×10^6^ µm^2^ endocervical tissue per study individual by using the computerized image analysis system, Quantimet, Q 550 IW (Leica) and Adobe Photoshop CS3 (Adobe Systems Incorporated, San Jose, CA).

## Results

### Distribution of CD4+ and antigen-presenting cells in the human endocervix

**Figure 1:**
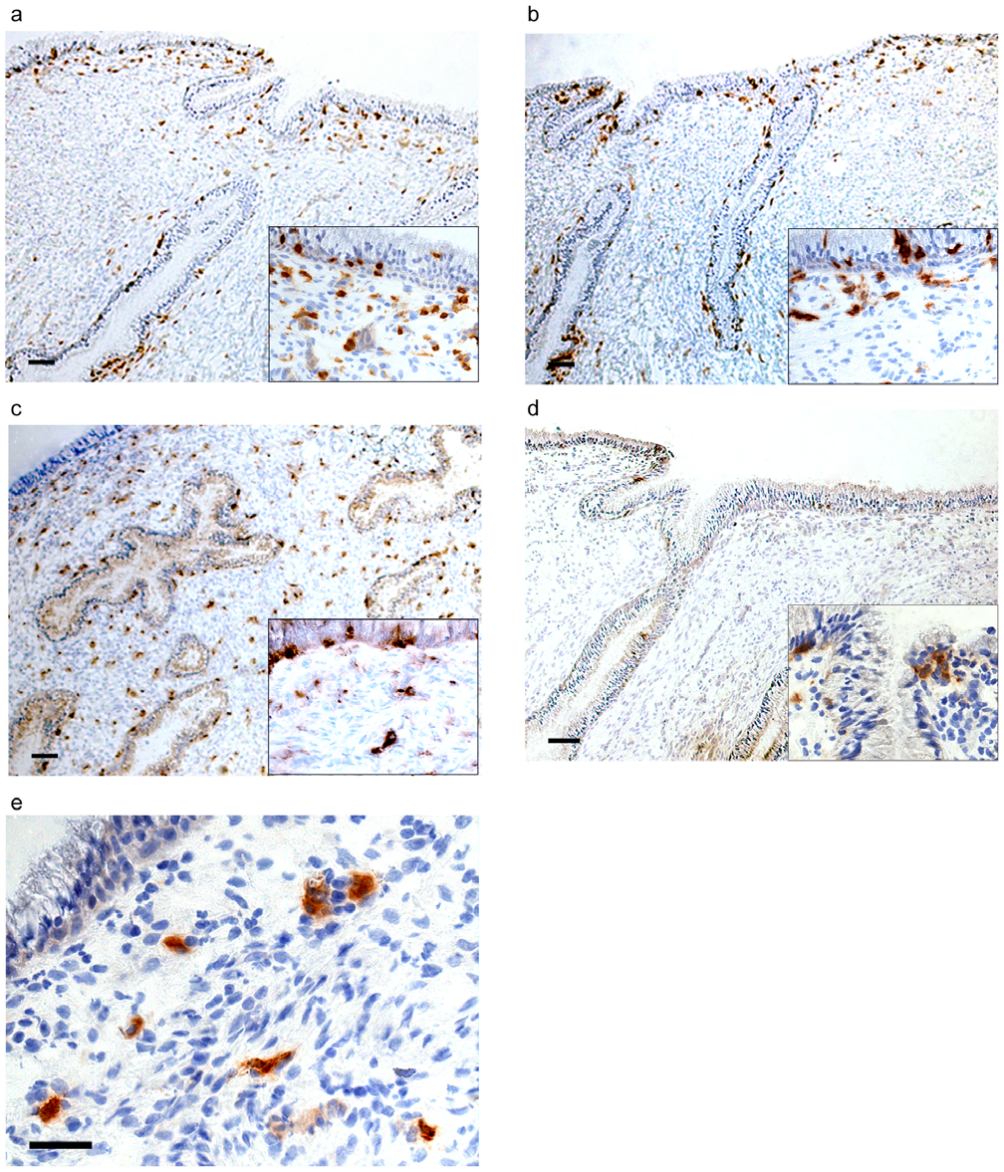
Distribution of CD4+ and antigen-presenting cells in the human endocervix. a: CD4+, Scale bar: 100 µm. Box depicts zoomed area proximal to columnar epithelium. b: CD11c+, Scale bar: 100 µm. Box depicts zoomed area proximal to columnar epithelium. c: CD68+, Scale bar: 100 µm. Box depicts zoomed area proximal to columnar epithelium. d: CD1a+, Scale bar: 100 µm. Box depicts zoomed area proximal to columnar epithelium. e: CD123+, Scale bar: 50 µm.

CD4+ cells were organized within or in close proximity to the endocervical columnar epithelium in all study subjects (median: 0.012 positive cells/100 µm^2^ [range: 0.006–0.027 positive cells/100 µm^2^]). Representative results are presented in Figure 1. CD11c+ cells (defined here as mucosal myeloid DCs) followed a similar distribution pattern and frequency as CD4+ cells (median: 0.013 positive cells/100 µm^2^ [range: 0.007–0.024 positive cells/100 µm^2^]) (Figure 1). CD68+ cells (defined here as macrophages) were also present in both the intraepithelial and submucosal compartments, but not in the clustered distribution noted for CD4+ and CD11c+ cells (Figure 1). However, CD68+ cells were the most frequent of all cellular markers investigated in this endocervical tissue (median: 0.036 positive cells/100 µm^2^ [range: 0.017–0.049 positive cells/100 µm^2^]), whereas CD1a+ cells (defined here as LCs), which were mainly located within the epithelium, were the least abundant (median: 0.002 positive cells/100 µm^2^ [range: 0.0004–0.014 positive cells/100 µm^2^]) (Figure 1). CD123+ cells were present in low frequencies and solely in the submucosal compartment (median: 0.007 positive cells/100 µm^2^ [range: 0.003–0.011 positive cells/100 µm^2^]) (Figure 1).

**Figure 2:**
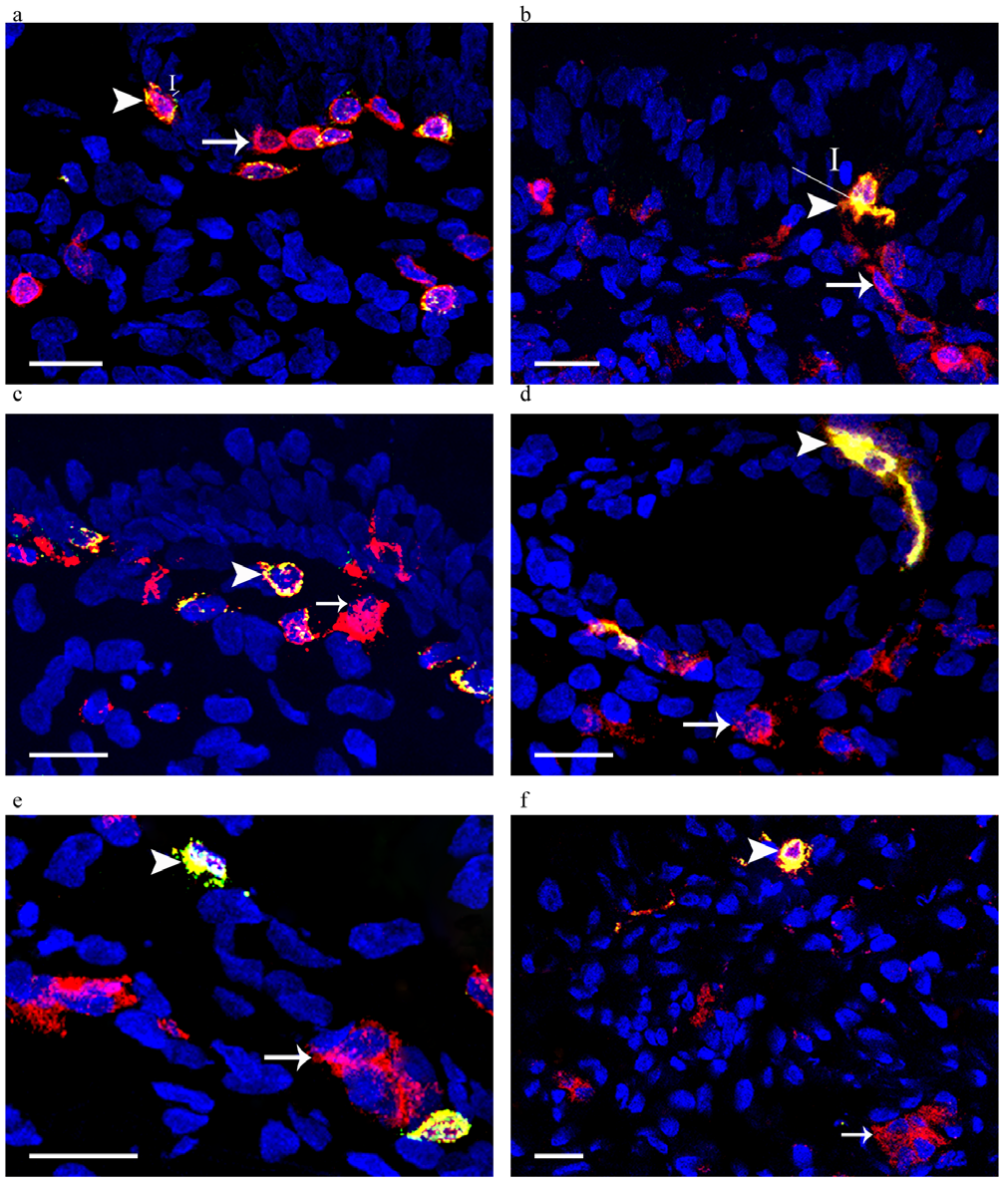
CD4 expression is present on T cells and antigen-presenting cell subsets. a: CD4+ (red),CD3+ (green), CD4+CD3+ (yellow), I: 3 µm, Scale bar: 25 µm. b: CD4+ (red), CD1a+ (green), CD4+CD1a+ (yellow), I: 24 µm, Scale bar: 25 µm. c: CD4+ (red), CD11c+ (green), CD4+CD11c+ (yellow), Scale bar: 25 µm. d: CD11c+ (red), CD1a (green), CD11c+CD1a+ (yellow), Scale bar: 25 µm. e: CD123+ (red), CD4+ (green,) CD123+CD4+ (yellow), Scale bar: 25 µm. f: CD123+ (red), BDCA-2+ (green,) CD123+BDCA-2+ (yellow), Scale bar: 25 µm. Arrows indicate single positive cells; arrowheads indicate double positive cells.

CD4 was expressed on 40–52% of endocervical CD3+ T cells, but also on all detected CD1a+ and CD11c+ cells. Representative results are presented in figure 2. Although all CD1a+ LCs were CD11c+, merely 15% of CD11c+ cells expressed CD1a (Figure 2). CD4 was also expressed on 8% of CD123+ cells (Figure 2). Since CD123 is expressed by plasmacytoid DCs (pDCs) but may also be expressed by additional cell types, a supplementary pDC-specific marker, BDCA-2, was used. Only 17% of the CD123+ cells co-expressed BDCA-2 and could thus accurately be defined as pDCs (Figure 2). The CD123+BDCA-2+ cells expressed neither CD1a nor CD11c (data not shown).

### CLRs and CCR5 are expressed in the human endocervix

**Figure 3:**
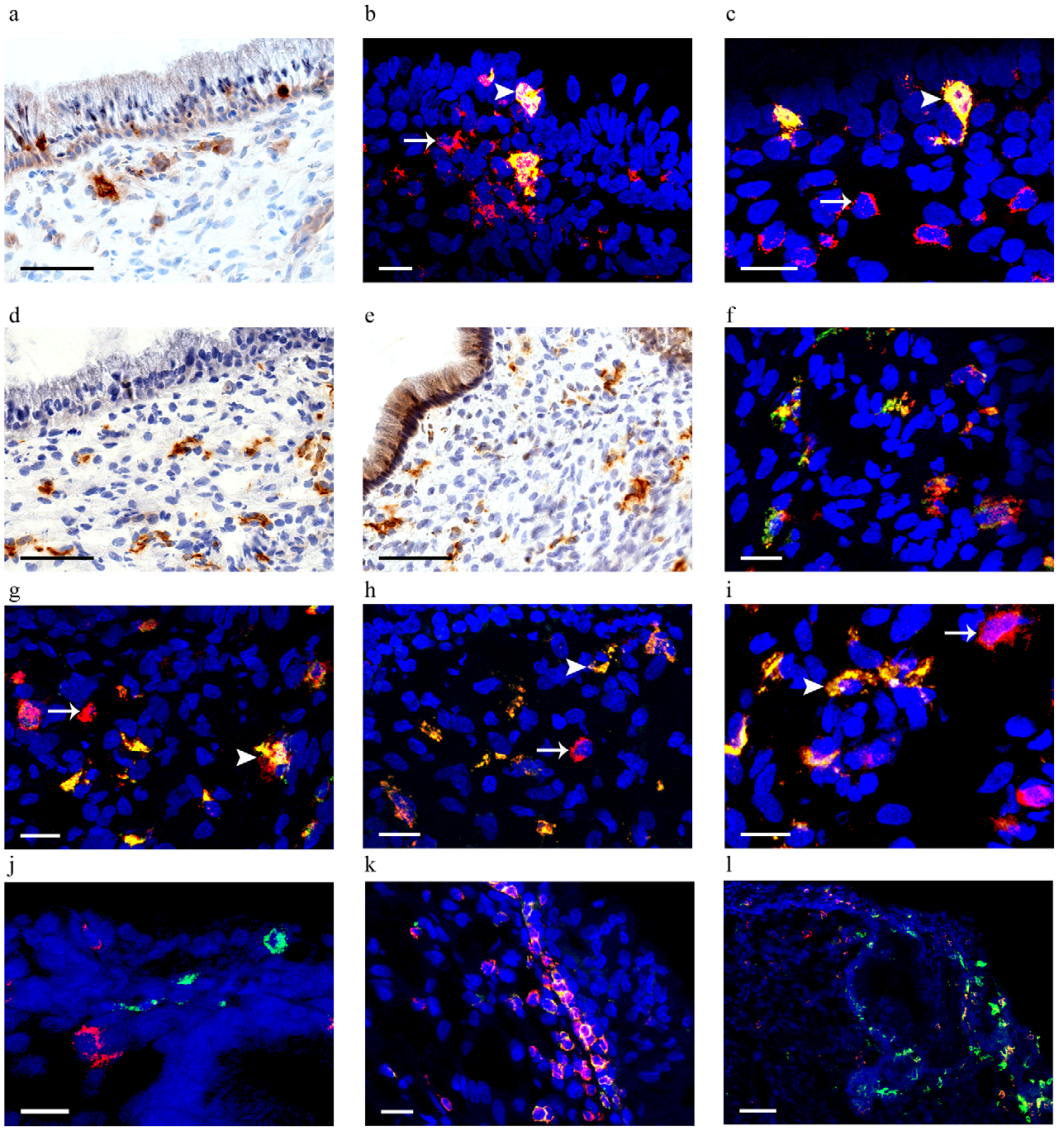
HIV-binding CLRs and CCR5 are expressed in the human endocervix. a: Langerin+, Scale bar: 100 µm. b: CD11c+ (red), Langerin+ (green), CD11c+ Langerin+ (yellow), Scale bar: 25 µm. c: CD4+ (red), Langerin+ (green), CD4+ Langerin+ (yellow), Scale bar: 25 µm. d: MR+, Scale bar: 100 µm. e: DC-SIGN+, Scale bar: 100 µm. f: MR+ (red),CD68+ (green), MR+CD68+ (yellow), Scale bar: 25 µm. g: DC-SIGN+ (red), CD68+ (green), DC-SIGN+CD68+ (yellow), Scale bar: 25 µm. h: MR+ (red), CD11c+ (green), MR+CD11c+ (yellow), Scale bar: 25 µm. i: DC-SIGN+ (red), CD11c+ (green), DC-SIGN+CD11c+ (yellow), Scale bar: 25 µm. j: CD1a+ (green), CCR5+ (red), Scale bar: 25 µm. k: CD4+ (green), CCR5+ (red), CD4+CCR5+ (yellow), Scale bar: 25 µm. l: CD11c+ (green), CCR5+ (red), CD11c+CCR5+ (yellow), Scale bar: 100 µm. Arrows indicate single positive cells; arrowheads indicate double positive cells.

Langerin+ cells were scarce (median: 0.009 positive cells/100 µm^2^ [range: 0.004–0.015 positive cells/100 µm^2^]) and mainly located within the epithelium but also in the underlying submucosa. Representative results are presented in figure 3. Langerin was expressed on 45% of the CD11c+ cells, 4% of the CD4+ cells (Figure 3) and all detected endocervical CD1a+ cells but not on CD68+ cells (data not shown). MR was the most frequent CLR detected (median: 0.05 positive cells/100 µm^2^ [range: 0.013–0.08 positive cells/100 µm^2^]). MR expressing cells were not detected in the epithelium; however, the CLR was present in the underlying submucosa as close as 30 µm from the epithelial surface (Figure 3). The distribution of DC-SIGN+ cells was similar to that of MR+ cells (as close as 50 µm from the epithelial surface) although less frequent (median: 0.009 positive cells/100 µm^2^ [range: 0.005–0.028 positive cells/100 µm^2^]) (Figure 3). Moreover, in some study individuals certain parts of the columnar epithelium expressed a weak unspecific DC-SIGN staining. Both MR and DC-SIGN were frequently expressed on CD68+ cells (97% and 35%, respectively) and on CD11c+ cells (86% and 38%, respectively) (Figure 3), but not on CD1a+ cells (data not shown). CD1a+ cells did not express CCR5, whereas CCR5+CD4+ cells and CCR5+CD11c+ cells were apparent within and adjacent to the endocervical epithelium (Figure 3). Neither of the investigated CLRs was present on CD123+BDCA-2+ cells (data not shown).

### Distribution of immune effector cells in the human endocervix

**Figure 4:**
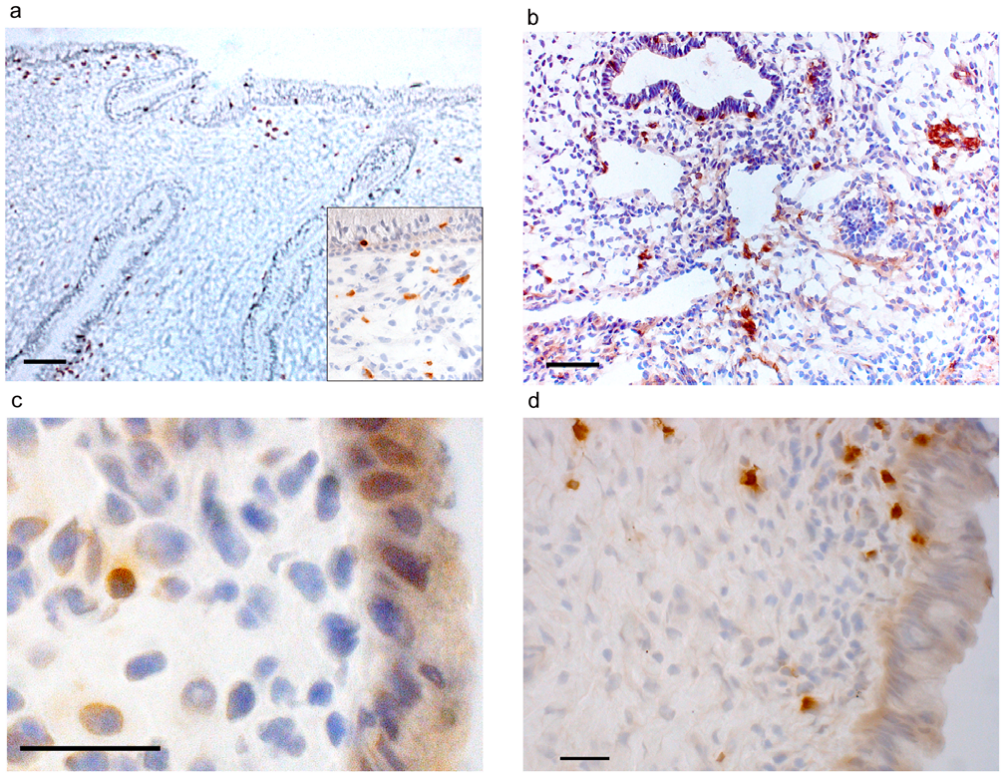
Endocervical distribution of immune effector cells. a: CD8+, Scale bar: 100 µm. Box depicts zoomed area proximal to columnar epithelium. b: CD56+, Scale bar: 100 µm. c: CD19+, Scale bar 50 µm. d: IgD+, Scale bar 50 µm.

Effector cells with potential cytotoxic activity or antibody-dependent functional activity include CD8+ cells (here defined as cytotoxic T cells), CD56+ cells (defined as a subset of natural killer cells) and CD19+ cells (defined as B cells), respectively. The distribution of CD8+, CD56+, CD19+ and IgD+ cells was characterized (Figure 4), but the cells were not further quantified.

Like the distribution pattern of CD4+ and CD11c+ cells, CD8+ cells were located within or close to the endocervical epithelium. CD56+ cells were also detected both in the epithelium and the submucosa. In contrast, CD19+ cells as well as IgD producing cells were only seen in the submucosa (as close as 120 µm and 55 µm to the epithelial surface, respectively).

## Discussion

Here, the presence of the potential HIV-binding receptors CCR5, Langerin, DC-SIGN and MR was visualized and their intraepithelial and/or submucosal localization in the human endocervix was demonstrated at the single cell level. These studies thereby complement previous data on the distribution of HIV receptors in human ectocervix [22] and endometrium [23]. Our present data from non-inflamed endocervical tissue indicate that Langerin+ cells are located in the single-cell layered epithelium in direct contact with the endocervical canal and could thus be accessible for HIV-binding and transmission. Furthermore, CCR5+ cells were seen in the endocervical epithelium and submucosa, corresponding to results from the human endometrium [23] and vaginal submucosa of rhesus macaques [24]. DC-SIGN+ and MR+ cells, although absent from the epithelial layer, were superficially located in the submucosa where other cell populations relevant for effector immune functions were found.

The relative contributions of human endocervical CD4+ T cells and antigen-presenting cells to initial HIV transmission are not clear, but experimental studies in the SIV-macaque model show focal infection of CD4+ T cells in the endocervical mucosa within a few days following intravaginal inoculation of SIV [25], [26]. Although intraepithelial CCR5+ CD4+ CD3+ T cells, CD1a+ and Langerin+ cells have previously been detected in cytobrush-obtained human cervical samples [14], [27], their in situ distribution has hitherto not been defined. The intraepithelial Langerin+ CD1a+ LCs were rare, but their location in the single-cell layered epithelium and their expression of Langerin and CD4 make them likely targets for primary HIV transmission. However, the direct sampling of viral particles by endocervical cells has not been shown in vivo. The ano-genital columnar epithelium has been proposed to lack LCs [28] and a previous study on human endocervices found no CD1a+ cells [19]. However,intraepithelial CD1a+ cells have been detected in endocervical tissue from rhesus macaques [16] and our group recently discovered Langerin+ CD1a+ cells in the single-layer uterine epithelium [23]. The cause of this discrepancy may be differing experimental procedures, underlying differences in the tissue or amount of tissue investigated. Furthermore, in contrast to previous findings in tissues from the human vagina [6] and cervix [27], we did not identify CD1a+ LCs that co-expressed CCR5.

Additional HIV target cells including DC-SIGN+ MR+ CD11c+ myeloid DCs and MR+ CD68+ macrophages were disseminated within the endocervical submucosa. The compartmentalization of MR+ and DC-SIGN+ expressing cells to the endocervical submucosa is similar to previous findings in human skin [29], endometrium [23] and ectocervix [22]. In a recent report using primary genital epithelial cells of cervical and endometrial origin, exposure to the HIV envelope glycoprotein was found to reduce the epithelial monolayer's integrity by up-regulation of inflammatory cytokines [30]. Thus, the underlying submucosal cells may theoretically be accessible to HIV-infected seminal fluid following sexual intercourse. Furthermore, the integrin CD11c together with the β chain CD18 form the complement receptor 4 (CR4 or CD11c/CD18). CR4 is expressed on antigen presenting cells including DCs and macrophages. Complement opsonized HIV can infect immature DCs through binding via complement receptor 3 (CR3) or CR4 and this has been shown to enhance HIV infectivity in vitro [31]. Thus, the CD11c+ cells in this study may bind HIV both via their CLRs as well as CR4. The endocervical mucosa is an immunologically active site including cells forming both innate and adaptive immune responses. A subpopulation of DCs, the BDCA-2+ CD123+ pDCs, forms an interface between innate and adaptive immunity, and these cells were found to be solely located in the submucosa. To our knowledge this is the first report on the presence of BDCA-2+ CD123+ pDCs in a non-inflamed human endocervix, although the number of such cells was very low. pDCs have been implicated as important players in both immune control of recurrent HSV infection [32] and the immune response following SIV exposure in the endocervix of rhesus macaques [25], but their function in the human endocervix remains to be established.

Among the additional leukocyte populations amenable to interaction with HIV are natural killer cells [33] and DC-SIGN expressing B cells [34]. However, the importance of these pathways for HIV-related immune events at different sites of the female genital mucosa needs further exploration. Indeed, natural killer cells express differing phenotypic markers throughout the female genital tract [35], and the CD56 marker used in the present study does not cover all these subtypes. Nevertheless, as reported previously [19], [36] also under non-inflammatory conditions, several types of immune cells including CD8+, CD56+ and CD19+ cells as well as antibody-producing cells were present and located near the CD4, CCR5 and CLR-expressing cells.

The expression of HIV target cells and receptors is critically dependent on the inflammatory status of the genital mucosa, as suggested by a recent report that described the persistence of HIV-receptor positive inflammatory cells in herpes-affected genital skin even long after clinical healing and clearance of herpes simplex virus (HSV) antigen [37]. Furthermore, female sex hormone levels including exogenous hormonal contraceptives affect the immune response in the female genital tract [38], [39], [40]. For example, CCR5 expression on uterine epithelial cells increases during the proliferative phase [41], whereas it remains relatively stable throughout the menstrual cycle in ectocervical epithelium [42]. Based on this and other findings, a “window of vulnerability” has been proposed in an interval of 7–10 days after ovulation during which susceptibility to HIV transmission increases due to hormonally induced immune suppression [43]. Hormone levels also influence localization of the border between the ectocervix and the endocervix. During puberty, pregnancy and hormonal contraceptive use, the endocervical epithelium may be exposed to the vaginal lumen: a condition called cervical ectopy [44]. When the integrity of the epithelial layer has been disrupted through mechanical microabrasion after intercourse or inflammatory conditions, cells in the submucosa of females with ectopy may be directly exposed to virus-containing seminal fluid. In fact, cervical ectopy has been associated with an increased risk of HIV infection [45]. The study subjects in this report represented three stages of the menstrual cycle, but the small size of this study group precluded the usefulness of statistical analysis of hormonal influence. However, the investigated markers were observed in endocervices representing all menstrual cycle stages.

The single-layer columnar epithelium of the endocervix is considered to make it a prime target site for sexual transmission of HIV. The detection at the single cell level enabled us to visualize the locations of the HIV-binding receptors in relation to one another, to plot their proximity to corresponding immune cells and to measure the distance of submucosal cells from the epithelial surface. An important application of our findings is that many of the current microbicide candidates are based on hindering the interaction of HIV with its receptors including the CCR5 and CLR molecules. Consequently, understanding susceptibility to HIV infection in non-inflamed human endocervical tissue during the relatively common situation of low-risk settings impacts the design of HIV receptor-blocking compounds. Future studies should also include subjects at higher risk of HIV infection such as those with inflammatory conditions and representatives of other ethnic and geographical settings.
